# Microbiome Engineering for Biotherapeutic in Alzheimer’s Disease Through the Gut–Brain Axis: Potentials and Limitations

**DOI:** 10.3390/ijms26115351

**Published:** 2025-06-02

**Authors:** Editha Renesteen, Jacqueline L. Boyajian, Paromita Islam, Amal Kassab, Ahmed Abosalha, Stephanie Makhlouf, Madison Santos, Hongmei Chen, Cedrique Shum-Tim, Satya Prakash

**Affiliations:** 1 Biomedical Technology and Cell Therapy Research Laboratory, Department of Biomedical Engineering, Faculty of Medicine and Health Sciences, McGill University, Montréal, QC H3A 2B4, Canada; editha.renesteen@mail.mcgill.ca (E.R.); jacqueline.boyajian@mail.mcgill.ca (J.L.B.); amal.kassab@mail.mcgill.ca (A.K.); stephanie.makhlouf@mail.mcgill.ca (S.M.);; 2 Pharmaceutical Technology Department, Faculty of Pharmacy, Tanta University, Tanta Al-Geish St., The Medical Campus, Tanta 31527, Egypt

**Keywords:** Alzheimer’s disease, gut–brain axis, probiotic, prebiotic, synbiotic, neuron

## Abstract

Alzheimer’s disease (AD) is a neurodegenerative condition characterized by considerable cognitive decline and functional impairment, primarily due to the progressive alteration of neurons, microglia, and astrocytes. Pathological manifestations of AD include the loss of synaptic plasticity, reduction in synaptic strength by amyloid-beta, aggregation, and neurotoxicity from tau protein post-translational modifications, all contributing to the disruption of neural networks. Despite its current pharmacological treatment for AD, different approaches to treat such disease are being developed, from a microbiome perspective. The microbiome encompasses a diverse microorganism, including beneficial bacteria that create a positive impact to diminish AD pathogenesis. Growing evidence suggests that probiotic, prebiotic, synbiotic, and postbiotics can positively modulate the gut–brain axis, reducing systemic inflammation, restoring neurotransmitter balance, and improving gut health, thereby possibly mitigating AD pathogenesis. Moreover, there is paraprobiotics as the most recently developed biotherapeutic with beneficial effects. This review explores the correlation between AD and gut–brain axis as a novel biotherapeutic target. The underlying mechanism of the microbiota–gut–brain axis in AD is examined. Novel insights into the current applications as potential treatment and its limitations are highlighted.

## 1. Introduction

**Figure 1:**
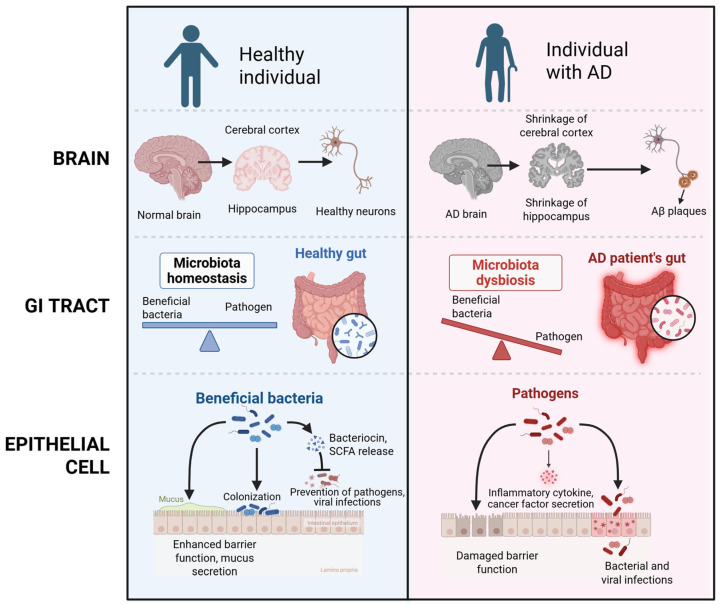
A comparison of brain, GI tract, and epithelial cells in healthy individual versus individual with AD. Healthy brain shows healthy neurons, while in AD it shows shrinkage of cerebral cortex and hippocampus, the formation of tau neurofibrillary tangle, and amyloid beta plaque. In healthy gut, there is microbiota homeostasis, while AD patients show dysbiosis and imbalanced microbiota. Epithelial cells of healthy individual show healthy epithelial cell and barrier integrity; however, there is a decrease in tight junction, increase in permeability, and inflamed epithelium in AD. The figure was created with Biorender.com.

Alzheimer’s disease (AD) is defined by a gradual deterioration of cognitive abilities and is associated to specific neuropathological features, including the presence of amyloid plaques and neurofibrillary tangles [1]. This disease is recognized as the primary contributor to dementia, which currently affects an estimated 24 million individuals worldwide, with projections indicating that this number will double in 2040 [2]. Even though the precise etiology of this condition remains unclear, this disease is frequently linked to the accumulation of amyloid beta (Aβ) peptides and tau proteins [3]. In patients with AD, there is notable atrophy of the cerebral cortex and hippocampus when compared to healthy brains. Furthermore, the neurons in AD brain form tau neurofibrillary tangle and Aβ plaques [3]. The comparison of healthy individuals and individuals with AD are described in Figure 1. Given the rising prevalence of AD, there is an urgent demand for effective therapeutic interventions. Currently, treatment strategies have focused on alleviating symptoms; however, recent research has begun to investigate the role of the gut–brain axis (GBA) in the pathogenesis of AD [4]. GBA is a bidirectional communication network that connects the central nervous system (CNS) with the gastrointestinal system, involving neural, endocrine, immune, and humoral mechanisms [5]. The gut microbiota, a critical component of this axis, has attracted considerable interest owing to its role in neuroinflammatory processes, metabolic functions, and immune regulation, all of which are essential in the relation of neurodegenerative diseases [6].

Emerging research highlights the significance of GBA in the pathophysiology of AD through the microbiota–gut–brain axis (MGB) in AD’s progression [7]. Exploring the MGB axis within the framework of AD could lead to the development of novel preventive and therapeutic approaches, including the use of probiotics, prebiotics, synbiotics, and precision medicine [2,3]. A significant method involves the application of probiotics, which are living microorganisms that provide health advantages by reestablishing the balance of gut microbiota. Evidence suggests that probiotics may alleviate symptoms associated with AD by modulating inflammation, enhancing gut barrier function, and producing neuroactive compounds that positively influence brain activity [8]. Prebiotics, non-digestible fibers facilitating the proliferation of beneficial gut microbiota, can modulate gut immunity and inflammation by enhancing the synthesis of short-chain fatty acids (SCFAs). SCFAs like butyrate are essential components in the pathogenesis of AD [8]. Preliminary studies in animal models suggest that prebiotics may enhance cognitive function and reduce neurodegenerative changes. The combined application of probiotics and prebiotics, referred to as synbiotics, has the potential to offer additional therapeutic benefits by enhancing gut health and its subsequent impact on cognitive performance [9].

Postbiotics, bioactive substances generated by probiotics during fermentation, present a promising approach for therapeutic intervention. These metabolites, which encompass SCFAs, polyphenols, and neurotransmitter precursors, can significantly influence neuronal health by mitigating oxidative stress and neuroinflammation [10]. Finally, the most recent biotherapeutic approach is paraprobiotics, inactivated microbial cells which can beneficially influence a host’s immune response via the adaptive and innate immune system [11,12]. This review aims to understand the relationship between AD and GBA as a novel therapeutic target. Specifically, the potential mechanism underlying MGB in AD is extensively discussed. Moreover, its current applications in animal research and human clinical trials are reviewed. Overall, the exploration of the MGB’s role in AD offers a potentially beneficial pathway for therapeutic intervention, and recent discoveries will be comprehensively analyzed.

## 2. Alzheimer’s Diseases, Gut–Brain Axis, and Microbiome

### 2.1. Pathophysiology of Alzheimer’s Diseases

Alzheimer’s disease (AD) represents a neurodegenerative disorder marked by a progress deterioration in cognitive abilities, diminished capacity for daily activities, and various psychiatric manifestations [13]. Initially, impaired memory is typically the initial symptom noticed, yet non-memory cognitive symptoms also develop as the disease progresses. For instance, families of AD patients often observe symptoms of disorientation and visual memory disturbance [14]. Moreover, comorbidity burden has been associated with deterioration in cognition, daily functioning, and neuropsychiatric symptoms, particularly in short-term studies [13]. Furthermore, genetic factors have a substantial impact on AD development; for instance, mutations in the amyloid precursor protein (APP) and presenilin proteins (PSEN1; PSEN2) are associated with early onset AD, whereas the apolipoprotein E (APOE-ε4) allele has been correlated with an increased risk of developing late-onset AD. Recent studies analyzing the entire genome have discovered more than 20 genetic loci linked to late-onset AD [15]. The formation of amyloid beta (Aβ) peptides and tau protein is fundamental to AD pathology, with various Aβ species, such as Aβ4-42 and pyroglutamate Aβ peptides possibly important in disease progression [3].

As the disease progresses, multiple factors, including oxidative stress, mitochondrial dysfunction, neuroinflammation, and insulin resistance are involved [7,16]. Notably as presented from initial stages, oxidative stress appears to be an important factor connecting various pathogenic pathways, contributing significantly to disease progression [17,18] and resulting in cognitive impairment through its effects on neurotransmitter and synaptic functions [19]. Furthermore, reactive oxygen species (ROS) targets multiple cellular components, including DNA, lipids, proteins, and calcium homeostasis, exacerbating mitochondrial dysfunction and creating a vicious cycle [19]. Aβ peptide directly generates ROS, while activated microglia produces neurotoxic factors that damage neurons [16]. Mitochondrial dysfunction in AD manifests through impaired energy production, increased oxidative stress, and disrupted pyrimidine synthesis, affecting neuronal function and viability [9,10]. Mitochondrial abnormalities in AD include alterations in biogenesis, dynamics, axonal transport, and interactions with the endoplasmic reticulum [20,21]. The mitochondrial dysfunction results in decreased ATP generation, enhanced ROS, and altered pyrimidine synthesis, affecting neuronal function and viability [22]. Interestingly, this dysfunction may precede and potentially drive AD pathology, challenging the amyloid cascade hypothesis and suggesting a primary mitochondrial cascade [22].

Neuroinflammation is a pivotal factor in AD pathogenesis, complementing the amyloid and tau hypotheses [23]. Key inflammatory mechanisms involve the activating microglia and astrocytes, which secrete pro-inflammatory cytokines and ROS, leading to neuronal damage [24]. Several pathways are implicated, including NF-κB, NLRP3/caspase-1, TREM2, and cGAS-STING [23]. Moreover, aging emerges as a critical risk factor for AD, as it is associated with persistent inflammation in both peripheral tissues and the central nervous system. Aβ accumulation further enhances neuroinflammation, accelerating AD progression [25]. Altogether, the complexity of AD pathogenesis suggests that single-target therapies may be insufficient, and multiple approaches addressing oxidative stress and other key mechanisms may be necessary for effective treatment [18].

### 2.2. Gut–Brain Axis

**Figure 2:**
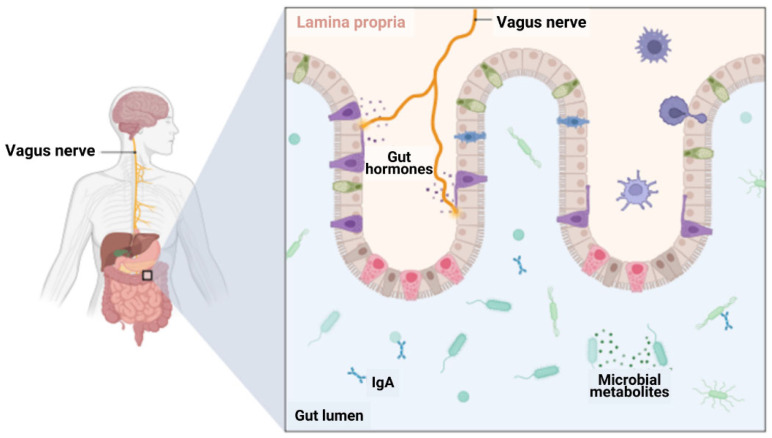
Gut–brain axis communication. Vagus nerve connects the brain and gastrointestinal tract. This figure is created with Biorender.com.

The gut–brain axis (GBA) constitutes a bidirectional communication network that connects the central nervous system (CNS) with the gastrointestinal system, involving neural, endocrine, immune, and humoral mechanisms [5]. This reciprocal interaction between the gastrointestinal tract and the CNS is mediated by various mechanisms, including the immune system, the vagus nerve, the enteric nervous system, and microbial byproducts SCFA [26], alongside the hypothalamic–pituitary axis and the gut microbiome (Figure 2). Microbiota significantly impact brain development and function through diverse signaling pathways [27]. In fact, five emerging hallmarks characterize the GBA: indistinguishability, emergence, bidirectional signaling, critical window fluidity, and neural homeostasis [27]. Thus, the gut microbiome is essential for preserving homeostasis within the CNS, as it regulates immune responses and synthesizing molecules that affect both the nervous and endocrine systems [5].

Importantly, dysbiosis within the gut microbiota is significantly associated with a range of CNS disorders, such as Alzheimer’s disease, Parkinson’s disease, and Amyotrophic Lateral Sclerosis (ALS), in addition to functional gastrointestinal disorders, including irritable bowel syndrome (IBS). Moreover, gut dysbiosis is linked to the production of bacterial amyloids and lipopolysaccharides, which can provoke immune responses and neuroinflammation [16]. Considering recent findings, studies increasingly underscore the critical importance of the gut microbiome in relation to brain health and associated disorders. In particular, the microbiome–gut–brain axis (MGB) integrates gut and central nervous system activities, influencing various neurological and psychiatric conditions. Emerging evidence also indicates that microbiota composition affects neurodevelopmental and neurodegenerative conditions [28]. Consequently, microbiota dysregulation has been observed in these disorders and modulating the gut microbiome may alter the severity of main pathologies [29]. As a result, the gut microbiota are thought to affect brain development, nociception, and complex host behaviors [30]. Moreover, emerging evidence suggests that strategies targeting the microbiome, such as prebiotics, probiotics, or dietary modifications, may offer promising avenues for alleviating symptoms associated with mood disorders, neurodevelopmental issues, and neurodegenerative diseases [29].

### 2.3. Microbiome Alterations in Alzheimer’s Disease

**Figure 3:**
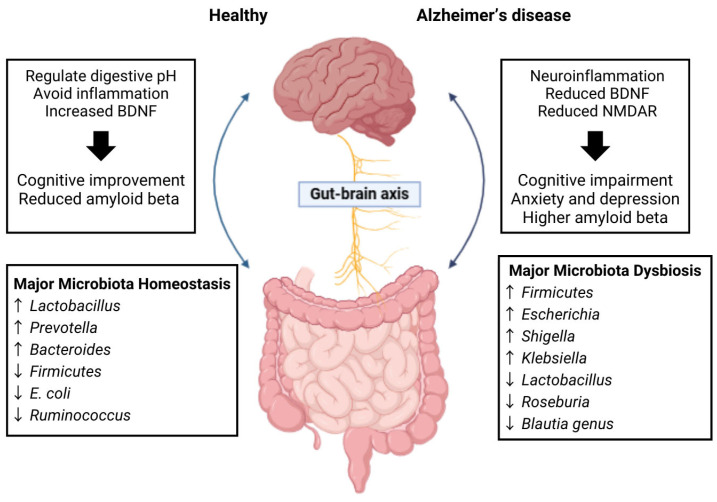
Microbiome in healthy and AD gut. *Lactobacillus*, *Prevotella*, and *Bacteroidetes* are enriched in healthy gut, while *Firmicutes*, *E. coli*, and *Ruminococcus* are not. In AD gut, *Firmicutes*, *Bacteroides*, *Escherichia*, *Shigella*, and *Klebsiella* are enriched in AD gut, while *Lactobacillus*, *Roseburia*, and *Blautia* genus are not. This figure is created with Biorender.com.

Alterations in gut microbiome have been implicated as a significant factor in the development of AD. Specifically, studies indicate a decrease in microbial diversity and significant changes in the composition of the gut microbiota of individuals diagnosed with AD, often marked by an elevation in *Firmicutes* and *Bacteroidetes* [31] (Figure 3). Comparable alterations have been observed in animal models of AD, where diminished levels of SCFAs and the formation of amyloid plaques in the intestines have been reported [32]. Given these findings, the MGB may play a pivotal role in the development of AD, as it has the capacity to influence brain function and cognitive processes. Furthermore, disturbances in gut microbiota may contribute to increased permeability of both the intestinal and blood–brain barriers, potentially exacerbating neurodegenerative processes. These insights imply that AD may be linked to gut health, suggesting that interventions aimed at modifying the gut microbiota through the introduction of beneficial microbial strains could represent innovative therapeutic avenues for AD [32]. Additionally, alterations in gut microbiome composition during AD progression also led to accumulation of specific amino acids, promoting neuroinflammation through T helper 1 cell activation and M1 microglia stimulation [33]. Such alterations have been documented in both AD mouse models and human subjects exhibiting mild cognitive impairment. In this way, gut microbiota dysbiosis may influence AD pathogenesis through the GBA, affecting Aβ oligomers, tau aggregates, and neuroinflammation. Overall, changes in gut microbiota composition, along with increased intestinal permeability and compromised blood–brain barrier integrity, are significant contributors to neuroinflammation and the onset of AD [34].

## 3. Current Treatment of Alzheimer’s Disease and Its Limitations

### 3.1. Current Treatment of Alzheimer’s Disease

Current treatments for AD primarily focus on symptom management using cholinesterase inhibitors and anti-glutaminergic (Table 1) [27,28]. Recently, the FDA-approved drug formulation is the monoclonal antibody donanemab, which has added to the available options. These medications generally provide modest cognitive enhancement and reduce functional decline. In addition to medications, nonpharmacological interventions, such as psychoeducation and behavioral strategies, play a crucial role in comprehensive care for AD patients [35]. Meanwhile, research efforts are increasingly focused on developing disease-modifying therapies targeting Aβ and tau pathologies, along with other pathways implicated in AD progression [27,28]. Promising approaches include secretase inhibitors, aggregation inhibitors, and drugs designed to enhance Aβ and tau clearance. Additionally, lifestyle interventions are being explored for disease prevention [36]. Interestingly, recent studies suggest that current symptomatic treatments may also influence amyloid precursor protein (APP) processing and tau phosphorylation, hinting at more complex mechanisms of action than previously understood.

### 3.2. Limitations of Current Treatment

Existing pharmacotherapy for AD exhibits limited effectiveness and primarily focuses on symptom control [36]. Currently, the main treatments consist of cholinesterase inhibitors and the NMDA receptor antagonist memantine, which offer temporary cognitive benefits but do not halt disease progression. Moreover, individual responses to these medications vary, and their effectiveness may decrease over time [40]. Although the pursuit of disease-modifying therapies has proven to be difficult, clinical trials in this area have largely produced disappointing results [35]. Today’s research efforts target underlying pathological processes including Aβ accumulation and tau deposition, antioxidants, and anti-inflammatory agents are being investigated as potential therapeutic options. Nonetheless, one major challenge in effectively managing AD remains the limited understanding of its underlying pathology, which continues to hinder treatment breakthroughs [41].

## 4. Mechanisms of AD via the Gut–Brain Axis

### 4.1. Gut Microbiota Imbalance (Dysbiosis)

The gut microbiota engages with the brain through various pathways, including neural, immune, endocrine, and metabolic mechanisms, thereby affecting brain homeostasis [42]. However, changes in the composition of the microbiota can result in increased intestinal permeability and a weakened blood–brain barrier. This disruption may trigger neuroinflammation and contribute to the characteristic features of AD like amyloid-beta accumulation, oxidative stress, and immune system dysregulation [7]. Pathogenic bacteria or their components, including lipopolysaccharides (LPS), can migrate into the bloodstream through a permeable gut, resulting in systemic inflammation. A previous study in 5×FAD mouse models demonstrated alterations in gut microbiota, correlated with an increased expression of the gut NLRP3 inflammasome and IL-1β in the gut [43]. Consequently, there is a growing focus on the role of gut microbiota in the development of neuroinflammation and neuronal damage [16]. Additionally, Toll-like receptors (TLRs) are pivotal in detecting microbial pathogens and initiating inflammatory responses, which may influence the progression of AD [44]. Neuroinflammation is subsequently mediated by astrocytes and microglia in response to pathological signals from altered microbiota [42].

### 4.2. Inflammation Modulation and the Immune System Activation

Systemic and intestinal inflammatory processes are increasingly recognized for their significant impact on brain pathology, with peripheral inflammation identified as a harmful contributor to the progression of AD [45]. Alterations in the composition of gut microbiota can disrupt the integrity of the blood–brain barrier, consequently promoting neuroinflammation and neuronal degeneration. Additionally, dysbiosis can lead to the release of proinflammatory cytokines, which may generate neuroinflammation and subsequent neurodegeneration in AD [46]. Furthermore, microbial metabolites, including LPS and bacterial amyloids, are capable of initiating immune responses within the CNS, resulting in neuroinflammatory processes [16].

Neuroinflammation involves both innate immune cells and an adaptive immune system [47]. Genome-wide association studies have identified specific genes linked to AD that are correlated to immune responses and the functioning of microglia. Notably, CD33 and TREM2 are implicated in neuroinflammatory mechanisms and are considered potential targets for therapeutic intervention [48]. Glial cells, especially astrocytes and microglia, are key mediators of neuroinflammation and neurodegeneration triggered by pathological changes associated with altered microbiota [42]. Once inflammatory mediators penetrate the brain, they activate microglial cells, which serve as the brain’s primary immune defenders. The activation of microglia, coupled with the infiltration of peripheral immune cells, has the potential to disturb the homeostasis of the CNS, increasing the likelihood of AD due to systemic inflammation [49]. Moreover, prolonged microglial activation increases the concentration of pro-inflammatory cytokines, exacerbating inflammation in the CNS. This buildup of pro-inflammatory cytokines in the brain is significantly linked to the formation of beta-amyloid plaques and the hyperphosphorylation of tau, characteristic features of AD pathology [42].

The gut microbiota plays a significant role in immune modulation by enhancing the production and functionality of regulatory T cells (Tregs). These cells are crucial for sustaining immune balance and preventing chronic inflammation. Tregs are pivotal in inhibiting pro-inflammatory responses and promoting an anti-inflammatory effect in the CNS. Furthermore, gut bacteria stimulate the secretion of anti-inflammatory cytokines like IL-10, which helps to suppress detrimental brain inflammation, enhancing neuroprotection [42]. Toll-like receptors have a complex role in AD, as they detect microbe-derived pathogens and initiate inflammatory responses [44]. In this context, LPS and various microbial metabolites can activate immune cells, including macrophages and monocytes, which subsequently release pro-inflammatory cytokines such as IL-6, TNF-α, and IL-1β. Additionally, chronic systemic inflammation can disrupt the integrity of the blood–brain barrier, facilitating the entry of peripheral inflammatory mediators and immune cells into the CNS [44].

Evidence suggests that probiotics, prebiotics, synbiotics, and postbiotics have been shown to exert positive effects on neuroinflammation and cognitive performance, particularly in the context of AD and obesity-induced insulin resistance. These interventions positively influence the gut microbiome, potentially reducing systemic and neuroinflammation through the GBA [50]. Moreover, synbiotic-derived metabolites, such as polyphenols show potential in mitigating AD-related neuropathologies by inhibiting amyloid aggregation and tau fibril formation [51]. In studies involving obese, insulin-resistance rats, the administration of prebiotics, probiotics, and synbiotics lead to improvements in hippocampal plasticity, mitochondrial function in the brain, and a reduction in microglial activation, ultimately contributing to the restoration of cognitive abilities [50].

### 4.3. Impact on Metabolites Production

The communication between the gut and the brain is bidirectional and occurs through multiple pathways, including the immune system, the vagus nerve, and microbial metabolites such as SCFAs [26].

#### 4.3.1. Short-Chain Fatty Acids (SCFAs)

Short-chain fatty acids (SCFAs), metabolites produced by gut microbiota, have demonstrated considerable promise in providing neuroprotective properties in the context of AD. Bacterial-produced metabolites, especially acetate, have been shown to mitigate cognitive decline and diminish neuroinflammation in mouse models of AD. This occurs through the inhibition of microglia activation and suppression of the ERK/JNK/NF-κB signaling pathway [52]. Furthermore, these metabolites disrupt the aggregation of toxic soluble Aβ, a significant pathological hallmark of AD [53]. Under normal circumstances, a healthy gut microbiome synthesizes SCFAs, with butyrate playing a crucial role in exerting anti-inflammatory effects and promoting overall brain health. However, in states of dysbiosis, there is often a decrease in the production of beneficial SCFAs alongside an increase in detrimental metabolites that can exacerbate neuroinflammation. Research has indicated that long-term dietary supplementation with SCFAs in AD mouse models can improve cognitive function by reducing Aβ accumulation and tau hyperphosphorylation. Additionally, SCFAs facilitate the glutamate–glutamine shuttle between astrocytes and neurons, which may enhance neuronal resilience against oxidative stress [54]. This underscores the importance of maintaining a healthy gut microbiome for cognitive health.

Gut microbiota, especially the phyla *Bacteroidetes* and *Firmicutes*, play a crucial role in the fermentation of dietary fibers, leading to the production of SCFAs such as butyrate, propionate, and acetate. These SCFAs possess the ability to cross the blood–brain barrier and exhibit neuroprotective properties. In particular, butyrate is recognized for its strong anti-inflammatory effects, as it suppresses the release of pro-inflammatory cytokines in both the gastrointestinal tract and the CNS, mitigating neuroinflammation. Additionally, butyrate acts as an inhibitor of Histone Deacetylase (HDAC) activity, which facilitates the expression of neuroprotective proteins, enhances synaptic plasticity, and strengthens memory functions. This form of epigenetic regulation may offer protection to neurons against the detrimental effects of amyloid toxicity and oxidative stress [55]. Butyrate has been shown to stimulate the synthesis of Brain-Derived Neurotrophic Factor (BDNF), a vital neurotrophic factor essential for synaptic plasticity and the survival of neurons. Elevated levels of BDNF are linked to enhanced cognitive abilities and memory preservation, providing a protective effect against neurodegeneration associated with AD. Additionally, gut microbiota may facilitate the production of glial cell-derived neurotrophic factor (GDNF), another important neurotrophic factor that supports the viability and functionality of dopaminergic neurons. Consequently, neurotrophic factors, especially BDNF, are equipped for processes such as synaptic plasticity, learning, and memory, positioning them as promising candidates for the treatment of AD [55].

#### 4.3.2. Tryptophan

The gut microbiota play a crucial role in modulating tryptophan metabolism, which subsequently leads to the synthesis of bioactive compounds that can exhibit either neuroprotective or neurotoxic effects. In particular, the conversion of tryptophan through the serotonin pathway is associated with neuroprotective outcomes, while its metabolism via the kynurenine pathway can produce neurotoxic byproducts. In a healthy gastrointestinal environment, there is a balance proportion between neurotoxic and neuroprotective kynurenine metabolites. However, dysbiosis can disturb this balance, potentially leading to increased production of neurotoxic compounds. Conversely, restoring a healthy gut microbiome may enhance the production of neuroprotective kynurenines, such as kynurenic acid, which has the potential to mitigate excitotoxicity—a detrimental process implicated in AD [56]. Recent studies underscore the critical involvement of tryptophan metabolism and the kynurenine pathway in AD and other neurodegenerative conditions. For instance, meta-analytical findings indicate that levels of kynurenine pathway metabolites are altered in patients with Alzheimer’s, Parkinson’s, and Huntington’s diseases when compared to healthy controls, suggesting a role for the kynurenine pathway in the pathogenesis of these disorders [57]. Elevated levels of quinolinic acid, an agonist of the N-methyl-D-aspartate receptor, have been documented in various neurodegenerative diseases, potentially exacerbating neuroinflammation and oxidative stress [56]. In AD condition, increased ratios of kynurenine to tryptophan and kynurenine to serotonin have been linked to inflammatory markers, cognitive decline, and adverse emotional states. Moreover, these ratios also show correlations with gray matter atrophy in critical brain regions, as well as heightened amyloid and tau accumulation [58]. Overall, the complex interactions between inflammatory mechanisms, kynurenine pathway dysregulation, and Alzheimer’s pathology highlight the potential of kynurenine pathway metabolites as both diagnostic indicators and therapeutic targets in neurodegenerative diseases.

### 4.4. Impact on Neurotransmitters

Gut microbiota influence the production and modulation of neurotransmitters and their precursors in both enteric and the CNS. The communication of neurotransmitters within the MGB is facilitated by interactions with the immune system, the vagus nerve, and various microbial metabolites [26]. Research supports that probiotics can generate neuroactive substances such as serotonin, gamma-aminobutyric acid (GABA), and acetylcholine, all of which can influence host physiology and mental well-being. The gut microbiota can synthesize, regulate, and modulate several vital neurotransmitters for optimal brain function. However, dysbiosis observed in AD may disrupt the production or availability of these neurotransmitters, potentially exacerbating the condition. Variations in gut microbiota composition can influence neurotransmitter synthesis, consequently affecting the progression of AD [26]. Moreover, in AD, the formation of amyloid-β plaques and tau tangles interferes with the equilibrium of neurotransmitters, leading to alterations in receptor expression and an excessive synthesis of glutamate.

The interaction between neurons and microglia, facilitated by neurotransmitter receptors, are essential for sustaining brain homeostasis. Disruptions in this communication may play a role in the development of AD pathology [59]. Additionally, the relationship between peripheral immune cells, such as T cells and monocytes, and resident brain immune cells like microglia, significantly influences the progression of AD [60]. These observations highlight the critical role of the neuroimmune axis in AD, suggesting that therapeutic approaches targeting neurotransmitter systems may effectively address neurochemical imbalances and modulate immune responses in this condition [60]. Neurotransmitters have a profound effect on immune responses, especially in both innate and adaptive immunity [61]. Furthermore, gut microbiota can indirectly affect neurotransmitter levels by modulating immune responses, which subsequently influence neurotransmitter systems. Chronic inflammation linked to gut dysbiosis may interfere with normal neurotransmitter function. Pro-inflammatory cytokines, including TNF-α and IL-1β, can disrupt neurotransmitter systems by diminishing serotonin production, impairing synaptic plasticity, and hindering neurotransmission, all of which are vital for memory and cognitive functions in AD.

#### 4.4.1. Serotonin

Serotonin serves as a key factor in the bidirectional communication between the gastrointestinal system and the brain [62]. This metabolite has demonstrated potential in alleviating symptoms and reducing neuropathological features of AD since altered serotonin signaling is commonly linked with cognitive impairment and behavioral change in AD [63]. Significant alterations in the AD brain extend well beyond Aβ and tau pathology, with disruptions in the serotonergic neurotransmitter system emerging as one of the most notable neurochemical changes, contributing but not limited to emotional and cognitive dysfunction [63]. Furthermore, disruptions in gut-derived serotonin may worsen these challenges by reducing the brain’s capacity to regulate mood and cognition effectively. Any imbalanced serotonin level can have significant effects since serotonin is critical for mood regulation, cognition, and memory. Consequently, gut dysbiosis may hinder serotonin production or modify its signaling in the gut, which in turn could impair the serotonergic pathways in the brain. Approximately 90% of the body’s serotonin is synthesized in the gastrointestinal tract, where gut microbiota play a pivotal role in regulating serotonin levels by interacting with host cells and facilitating tryptophan metabolism. Recent studies emphasize the significant involvement of gut microbiota in the regulation of serotonin synthesis and homeostasis. Specifically, the gastrointestinal system stores the body’s serotonin, predominantly produced by enterochromaffin (EC) cells [64]. Research indicates that spore-forming bacteria within the gut microbiota enhance serotonin biosynthesis in colonic EC cells, influencing gastrointestinal motility and platelet activity. Interestingly, the microbiota’s effect on serotonin levels is rapid, with notable increases occurring within three days of exposure to specific pathogen-free microbiota in germ-free mice [65]. Furthermore, microbial metabolites substantially affect serotonin production, subsequently impacting a variety of host physiological functions. Importantly, adjustments in serotonergic activity have shown potential in reducing AD-related brain neuropathology and symptoms in preclinical investigations [63]. In addition, bacterial enzymes facilitate the generation of free serotonin in the gut lumen [65]. Overall, these findings highlight the critical role of host–microbiota interactions in the regulation of essential serotonin-related biological processes.

#### 4.4.2. Gamma-Aminobutyric Acid (GABA)

In healthy conditions, specific bacterial species contribute to gamma-aminobutyric acid (GABA), a neurotransmitter that exerts neuroprotective effects and plays a role in regulating brain activity by preventing excessive neuronal excitation. However, dysbiosis can have substantial impacts on GABAergic signaling, intestinal barrier function, and inflammation associated with AD [66]. Specifically, it may lead to decreased GABA synthesis, increasing neuronal excitability, and triggering stress response. In this context, GABA deficiency can lead to excitotoxicity, a condition characterized by the damage and death of overactive neurons which, in turn, accelerates neurodegenerative processes linked to AD. Furthermore, reductions in GABAergic signaling are frequently noted in individuals with AD, which is associated with memory impairment and cognitive deterioration.

Certain gut microbes, such as *Lactobacillus* and *Bifidobacterium*, are capable of synthesizing GABA, an inhibitory neurotransmitter that plays a crucial role in the regulation of stress, mood, and anxiety. Additionally, GABA may influence the integrity of the intestinal barrier, and the inflammation associated with AD [66]. Recent studies underscore the important contribution of gut microbiota to the production of GABA, a vital inhibitory neurotransmitter. For instance, *Bifidobacterium adolescentis* has emerged as a promising candidate for GABA production within the human gastrointestinal system, with in vivo investigations validating its capacity to enhance GABA synthesis [67]. In addition to *Bifidobacterium*, other notable GABA-producing bacteria include *Bacteroides* and various Lactic Acid Bacteria, especially *Lactobacillus* [66]. The synthesis of GABA by gut microbiota may, therefore, affect not only gut health but also vagus nerve signaling and GABAergic pathways–mechanisms often disrupted in neurological conditions such as AD [66]. This communication along the gut–brain axis involving GABA may be implicated in a range of mental health disorders, including anxiety, depression, and autism spectrum disorder [68]. Finally, a negative correlation has been observed between the abundance of fecal Bacteroides and brain activity patterns linked to depression, indicating potential therapeutic avenues for GABA-producing bacteria in the treatment of mental health issues.

#### 4.4.3. Dopamine

Dopamine is essential for motivation, reward mechanisms, and learning processes. Gut microbiota, including *Enterococcus* and *Escherichia* species, can synthesize dopamine, a neurotransmitter crucial for reward mechanisms and motivation, which are intrinsically linked to cognitive processes such as memory and learning. Recent studies, therefore, underscore the pivotal function of gut microbiota in modulating dopamine synthesis and signaling via the MGB. For instance, *Enterococcus faecium* has been demonstrated to produce dopamine when provided with its precursor, L-dopa [69]. Furthermore, the gut microbiome affects the bioavailability of dopamine through several pathways, including vagus nerve communication, interactions with the immune system, and the production of microbial metabolites [70]. Importantly, specific bacterial genera, such as *Prevotella*, *Bacteroides*, and *Lactobacillus*, have been associated with the regulation of dopamine levels. Additionally, *Bifidobacterium dentium* has the capacity to increase tyrosine concentrations in both fecal matter and the brain, which may influence the dopamine signaling pathway [71]. Given these findings, the gut microbiota’s role in the production and modulation of neurotransmitters, especially dopamine, may hold substantial implications for neurological conditions, including gliomas [72]. Alterations in gut microbial composition, however, may impact dopamine levels and receptor functionality, subsequently impacting cognitive abilities and memory in individuals with AD. Certain genera of gut bacteria, such as *Prevotella* and *Lactobacillus*, are also known to play a role in dopamine metabolism and bioavailability, both of which are vital for optimal brain performance [70]. Some gut bacteria can even synthesize dopamine precursors; however, dysbiosis may disrupt this regulation, potentially leading to dopamine deficiencies and impaired in AD.

#### 4.4.4. Acetylcholine

Choline serves as a key factor precursor for acetylcholine, a neurotransmitter essential for cognitive functions such as memory and learning. In AD, there is a notable decrease in acetylcholine levels, which contributes to cognitive deterioration. Although the precise mechanisms by which gut microbiota affect acetylcholine production are not fully understood, it is believed that dysbiosis may alter the processes of acetylcholine synthesis or degradation, potentially exacerbating cognitive deficits. The cholinergic hypothesis serves as a key framework in AD research. The loss of cholinergic neurons contributes to the symptoms of AD [73]. Furthermore, nicotinic acetylcholine receptors (nAChRs), particularly the α7 subtype, are thought to play a role in the pathogenesis of AD, due to their interactions with amyloid-β peptides [74]. Current therapeutic strategies for AD predominantly aim to enhance cholinergic signaling, with cholinesterase inhibitors being the primary treatment modality [73]. Nevertheless, emerging research is investigating innovative approaches that target nAChRs, especially the α7 subtype, as potential therapeutic options. Positive allosteric modulators of α7 nAChRs are being recognized as promising candidates for AD treatment, with the goal of rectifying the underlying receptor dysfunction [75]. Overall, understanding the interplay between gut microbiota and acetylcholine production could open new avenues for therapeutic interventions in AD.

### 4.5. Direct Vagus Nerve Communication

The gut microbiota are essential in the bidirectional communication between the gastrointestinal system and the CNS, a relationship referred to as the MGB. This interaction has significant implications for the pathophysiology of AD [26]. Specifically, this interaction is facilitated through multiple mechanisms, including the vagus nerve, the immune response, and microbial metabolites such as GABA [66]. Disruptions in GABAergic signaling and changes in gut microbial populations have been linked to the development of AD. Additionally, nitric oxide (NO) signaling and amyloid proteins derived from gut bacteria are also implicated in the pathogenesis of AD [66].

Nonetheless, comprehensive research, including extensive longitudinal clinical trials, is essential to clarify the underlying mechanisms and the therapeutic efficacy of targeting this axis in the management of AD [76]. Furthermore, the vagus nerve, which establishes a direct link between the gut and the brain, may represent an additional pathway through which gastrointestinal disturbances influence neuroinflammation. For instance, signals from gut inflammation could activate the vagus nerve, relaying pro-inflammatory messages to the brain. External vagus nerve stimulation has demonstrated potential in altering the morphology of microglia, which may facilitate a transition from a neurodestructive to a neuroprotective phenotype in models of AD [77]. In addition, the interaction between neurons and microglia, which is mediated by neurotransmitter receptors, plays a vital role in sustaining brain homeostasis; any disruption in this communication may exacerbate the progression of AD [59]. Overall, the MGB, the vagus nerve, and microbial metabolites have been associated with the pathophysiology of AD. Thus, modulating the vagus nerve, whether through stimulation or inhibition, may influence the release of neurotransmitters such as acetylcholine, serotonin, and GABA, thereby affecting cognitive functions and emotional regulation.

### 4.6. Neuroprotection Enhancement

Recent research has underscored the promising role of probiotics, prebiotics, and synbiotics in neuroprotection and neurogenesis. Specifically, these interventions have the capacity to influence the gut microbiome, thereby affecting the GBA and potentially alleviating the symptoms of neurodegenerative diseases such as AD and Parkinson’s disease [36,78]. Evidence indicates that probiotics and prebiotics can enhance cognitive function, diminish neuroinflammation, and promote neurogenesis [8]. For instance, a multi-strain probiotic formulation has been shown to confer neuroprotective benefits in a mouse model of acute inflammation, preventing pro-inflammatory cytokine increases and enhancing hippocampal neurogenesis [79]. Similarly, a synbiotic combination of polymannuronic acid and *Lacticaseibacillus rhamnosus* GG demonstrated neuroprotective properties in a mouse model of Parkinson’s disease, modulating dopaminergic neuronal survival and motor function [80].

In addition to these interventions, the endocannabinoid system (ECS) has been identified as a promising avenue for the treatment of AD. Research indicates that modulation of the ECS, particularly via the CB1 and CB2 receptors, can exert neuroprotective effects in models of AD by mitigating β-amyloid toxicity, tau phosphorylation, and neuroinflammatory responses. Furthermore, cannabinoids have shown potential in addressing various pathological mechanisms associated with AD, such as excitotoxicity, mitochondrial dysfunction, and oxidative stress [81]. Moreover, inhibitors targeting fatty acid amide hydrolase (FAAH) and monoacylglycerol lipase (MAGL) have demonstrated efficacy in reversing cognitive impairments and alleviating neuroinflammation in mouse models of AD [82]. Although preclinical findings are promising, clinical trials have largely concentrated on alleviating behavioral symptoms, including agitation and aggression [83]. Importantly, the gut microbiota have been shown to influence the ECS, complementing the regulation of immune responses, inflammation, and neural plasticity. Therefore, the activation of the ECS may confer neuroprotective benefits by diminishing neuroinflammation, fostering synaptic plasticity, and promoting neuronal survival. Ultimately, enhancing endocannabinoid signaling through modulation of the gut microbiota could potentially safeguard neurons from amyloid-induced damage and neuroinflammation, thereby decelerating the progression of AD [81].

### 4.7. Gut-Derived Antioxidants and Mitochondrial Protection

Recent studies underscore the potential role of gut-derived antioxidants in alleviating the pathology associated with AD through the protection of mitochondrial function. The progression of AD is significantly influenced by oxidative stress and mitochondrial impairment [84]. To address this issue, a variety of antioxidants, such as coenzyme Q10 and lipoic acid, have demonstrated efficacy in targeting mitochondrial sites and mitigating oxidative injury [85]. Importantly, indole compounds synthesized by gut microbiota possess neuroprotective, antioxidant, and anti-inflammatory characteristics, which may affect the onset and development of AD [86]. Furthermore, probiotic treatments, exemplified by SLAB51, have been shown to activate SIRT1-dependent pathways, thereby reducing oxidative stress in the brains of 3xTg-AD mice [87]. Moreover, mitochondrial dysfunction, characterized by reduced energy production and heightened oxidative stress, is a defining feature of AD. In this context, SCFAs and other metabolites generated by gut microbiota may enhance mitochondrial performance and safeguard neurons against oxidative damage, thereby promoting overall cellular health.

## 5. Biotherapeutic Strategies for Alzheimer’s Disease

### 5.1. Probiotics, Prebiotics, Synbiotics, Postbiotics, and Paraprobiotics

Probiotics are characterized as live microorganisms that provide health advantages when ingested in sufficient quantities. Meanwhile, prebiotics serve as substrates that are metabolized by host microorganisms, fostering the proliferation of beneficial bacterial populations [88]. A significant amount of research has been dedicated to exploring the relationship between probiotics, prebiotics, and the gut microbiota’s influence on cognitive functions. This work underscores the potential roles of probiotics, prebiotics, and dietary components on the development of brain and behavior through various mechanisms, including immune, neural, and metabolic pathways [52]. Notably, probiotics treatment alters neuropeptide abundance in the brain, correlating with changes in gut microbiome composition; in particular, the hippocampus demonstrated a pronounced sensitivity to probiotic interventions [89]. Common probiotic strains include *Bifidobacterium* and *Lactobacilli*, while prebiotics like fructooligosaccharides and inulin are frequently utilized [89].

International Scientific Association for Probiotics and Prebiotics (ISAPP) updated the definition of synbiotic as a mixture comprising live microorganisms and substrate selectively utilized by microorganisms that confers a health benefit on the host [9]. These formulations can be complementary or synergistic, with the latter requiring specific stimulation of the added microbe by the substrate to maximize their effects [90]. Previous research has indicated that synbiotics possess the potential to prevent and manage a range of diseases by reestablishing and recolonizing the intestinal microflora [91]. The combined use of probiotics and prebiotics in synbiotics has demonstrated improved viability of probiotics and enhanced health advantages, including immune modulation, cancer prevention, and management of inflammatory bowel disease (IBD) [92]. Collectively, probiotics, prebiotics, and synbiotics have shown potential in modulating immune response, preventing infections, managing IBD, and even contributing to cancer treatment. Their ability to alter gut microbiota and induce various host functions makes them promising candidates for next-generation therapeutics [93].

Postbiotics, metabolic byproducts produced by beneficial bacteria during fermentation or growth, have emerged as a relatively new concept in health and nutrition. The World Health Organization (WHO) identifies two primary categories of postbiotics as non-viable microbial cells and fermented infant formulae. The byproducts include SCFAs, enzymes, peptides, vitamins, organic acids, antimicrobial bacteriocins, exopolysaccharides, and teichoic acids [12,94]. Postbiotics offer advantages like enhanced stability and rapid activation in the intestine [94]. Moreover, they exert their effects on health through multiple mechanisms, such as modifying the gut microbiota composition, regulating the synthesis of microbial metabolites, and improving the integrity of the intestinal barrier [95]. Additionally, they also possess immunomodulatory, anti-inflammatory, antioxidant, and anti-proliferative effects. Importantly, the NF-κB and MAPK pathways have been identified as potential targets for the immune-modulating effects of postbiotics on host health [94].

The recent biotherapeutic approach of paraprobiotics, inactivated microbial cells, is reported to confer a health benefit to humans through the mechanism of modulating adaptive and innate immune systems and improving peristaltic movement [11,12]. Also, biotics have effects such as anti-inflammation, antioxidant, and antiproliferation [11]. Research has shown that paraprobiotics from the cell surface component of *Lactobacilli* play a vital role when interacting with the host cell. The initial contact between these components triggers subsequent physiological responses, acting as effector molecules in which beneficial effects are observed in the host [96].

**Figure 4:**
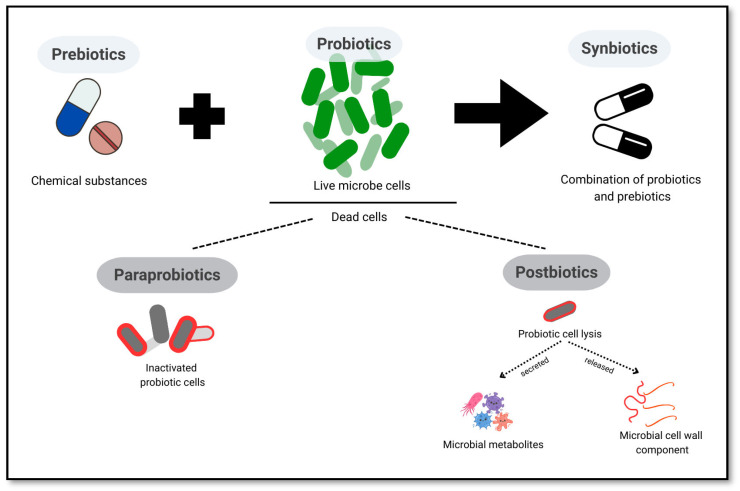
The differences between probiotics, prebiotics, synbiotics, postbiotics, and paraprobiotics.

Paraprobiotics for AD has not been tested in animal models and humans, yet the beneficial effects from this approach might be promising to decrease AD progression. While the mechanisms underlying most bioactivities remain unclear, scientific evidence indicates that postbiotics and paraprobiotics possess various functional properties, including interacting with diverse molecules or receptors through inhibitory actions, as well as contributing to the maintenance of host microbiota homeostasis [12]. The differences between biotherapeutic strategies of probiotics, prebiotics, synbiotics, postbiotics, and paraprobiotics are illustrated in Figure 4.

### 5.2. Evidence from Animal Studies

#### 5.2.1. Probiotics

Experimental in animal models are essential to understand AD pathophysiology despite its constraints in less accuracy to recapitulate clinical scenarios. Preclinical evidence studies in biotherapeutic strategies are summarized in Table 2.

Probiotics have been identified as modulators of inflammatory processes and oxidative stress by altering the gut microbiota. *Lactobacillus plantarum* DP189 has demonstrated efficacy in alleviating neurodegeneration linked to α-SYN accumulation in the substantia nigra of PD mice. This effect is attributed to its ability to reduce oxidative stress, inhibit pro-inflammatory responses, and modify the composition of gut microbiota [97]. Specifically, this bacterium has been shown to activate the nuclear factor erythroid 2-related factor (Nrf2)/ARE and Peroxisome proliferator-activated receptor-γ coactivator (PGC)-1α signaling pathways while suppressing the NOD-like receptor protein 3 (NLRP3) inflammasome [97].

The administration of *B. breve* MCC1274 has been shown to activate the protein kinase B (Akt)/glycogen synthase kinase-3β (GSK-3β) signaling pathway, potentially contributing to the observed reductions in presenilin1 levels and the suppression of tau phosphorylation [46,47]. *B. breve* MCC1274 could mitigate AD-like pathologies in wild-type mice by reducing Aβ42 levels, inhibiting tau phosphorylation, reducing neuroinflammation, and enhancing synaptic protein levels [98]. Furthermore, treatment with multi-strain probiotics has demonstrated improvements in cognitive deficits and pathological alterations in SAMP8 mice, addressing issues such as neural damage, Aβ and tau pathology, as well as neuroinflammation [99].

Another example is ProBiotic-4, consisting of *Bifidobacterium lactis*, *Lactobacillus casei*, *Bifidobacterium bifidum*, and *Lactobacillus acidophilus*, which has been found to significantly improve age-related dysfunctions in both the intestinal and blood–brain barriers. It also led to reductions in interleukin-6 and tumor necrosis factor-α at both mRNA and protein levels, alongside reductions in plasma and cerebral lipopolysaccharide (LPS) concentrations, Toll-like receptor 4 (TLR4) expression, and nuclear factor-κB (NF-κB) nuclear translocation within the brain [100]. Furthermore, *L. plantarum* DP189 altered the gut microbiota profile in PD mice by decreasing the prevalence of pathogenic bacteria such as *Proteobacteria* and *Actinobacteria* while enhancing the abundance of beneficial probiotics like *Lactobacillus* and *Prevotella* [97]. These findings collectively indicate that probiotic interventions targeting gut microbiota may provide therapeutic advantages in addressing microbiota–gut–brain (MGB) deficits and cognitive decline associated with aging. The mechanisms involved appear to include the inhibition of TLR4- and retinoic acid-inducible gene-I (RIG-I)-mediated NF-κB signaling pathways and inflammatory responses [100]. In the context of Alzheimer’s disease (AD), mice displayed reduced microbiota diversity and altered gut microbiota composition, with a significant correlation observed between microbiota richness and cognitive performance [101].

Previous work from de Rijke et al. (2022) provided evidence to decrease AD progression and beneficially affect the composition of gut microbiota, SCFA levels, and cognitive performance in AD models using probiotics derived from genera *Lactobacillus* and *Bifidobacterium* [102]. Specific strains like *Bifidobacterium longum* (NK46), *Clostridium butyricum*, and the mixture SLAB51 have shown particular promise as well [102]. In addition, new probiotics such as *Akkermansia muciniphila* and *Faecalibacterium prausnitzii* are being explored for their therapeutic potential in neurodegenerative disorders [103]. These findings underscore the significant influence of gut microbiota on neurological health and suggest that probiotics may serve as a promising therapeutic approach for cognitive deficits linked to neurodegenerative conditions [104].

Most of these animal-based probiotic intervention trials have observed some extent of beneficial effects on AD, although different study parameters were used, such as probiotic strains, dose, treatment duration, and subject of animal model. For example, oral administration of *Bifidobacterium breve* MCC1274 has been shown to enhance cognitive function in App^NL-G-F^ mice and individuals with mild cognitive impairment, leading to mitigation of AD-like pathologies. This probiotic treatment in wild-type (WT) mice led to a decrease in soluble hippocampal Aβ42 levels, which was associated with a reduction in presenilin1 protein levels and a decrease in phosphorylated tau levels [98]. Conversely, administering *L. plantarum* PS128 gave no significant alteration in cognitive dysfunction, Aβ, and tau levels, and cognition neuronal loss in 3xTg AD mice [105].

Several factors might contribute to observed heterogeneity in across studies. Strain specificity could be one of the most influential for such reason. Beck et al. (2022) reported the importance of probiotic strain specificity and its impact on influencing host–microbiome interaction in the human preterm gut [106]. Moreover, animal models also play a vital role in causing inconsistent results. As an example, almost no preclinical benefits were observed from *L. plantarum* PS128 consumption in 3xTg mice, while these benefits were shown in chemically construct diabetic animal models [105]. Finally, sexual differences seem to be another cause of divergent therapeutic effect between male and female animal models. For instance, VSL#3 supplementation was correlated to reduce AD pathophysiology in female mice, but not to male mice, indicating a sex-dependent probiotic effect [107].

#### 5.2.2. Prebiotics

Prebiotic fibers positively affect brain function through anti-inflammatory and epigenetic mechanisms [108]. This compounds have the capacity to alter the gut microbiome, thereby enhancing the synthesis of advantageous metabolites such as butyrate and indoles, which may regulate gut immunity and brain functionality [109]. Over the years, the effect of prebiotics on AD were investigated, including in inulin, fructooligosaccharide, xylooligosaccharide, and polyphenol which can maintain the health of gut microbiota.

One of the most studied prebiotic compounds is mannan oligosaccharides (MOS), a compound that mitigates the accumulation of amyloid-beta (Aβ) in various regions of the brain, including the cortex, hippocampus, and amygdala, lowering levels of corticosterone and corticotropin-releasing hormones, while simultaneously enhancing the expression of norepinephrine. MOS treatment has been effective in preserving the integrity of the gut barrier and preventing the leakage of lipopolysaccharides (LPS). In a study involving 5×FAD mice, MOS significantly reduced cognitive and mental deficits, a result that may be linked to alterations in the microbiome and elevation production of SCFAs in the gut [110].

A similar oligosaccharide that has been extensively studied is galactooligosaccharides (GOS) and fructo-oligosaccharides (FOS). This prebiotic demonstrated the most pronounced effect in reversing cognitive impairments in APP/PS1 mice, with a combination of FOS and GOS, whereas FOS alone exhibited no significant impact. Both GOS and the FOS-GOS combination influenced multiple biological targets, reducing pro-inflammatory cytokines like IL-1β and IL-6 and Aβ levels, as well as modulation of neurotransmitters level, including serotonin and GABA in the brain [111]. Moreover, FOS ameliorates cognitive impairment and alleviates Aβ accumulation in the brain of AD model mice. Additionally, FOS has been shown to improve cognitive function and reduce Aβ accumulation in AD mice models, providing protective effects against the disease through mechanisms such as the reduction in neuronal apoptosis and the mitigation of oxidative stress [112].

A closer examination of prebiotics studies indicates a variety in therapeutic effectiveness in AD models. For example, female mice with the *APOE4* genotype that were administered inulin demonstrated a restoration of alpha diversity and a significant reduction in *Escherichia coli*, along with diminished responses in inflammation-related pathways. However, when compared to the male animal model, the *APOE4* females exhibited lower metabolic responses, particularly in the levels of SCFA-producing bacteria and the relevant kinases, notably those associated with acetate and *Erysipelotrichaceae*. This heightened vulnerability of APOE4 females to AD, potentially attributed to suboptimal energy production, underscores the necessity of exploring precision nutrition strategies to address dysbiosis and mitigate AD risk in future research [113].

#### 5.2.3. Synbiotics

Many studies have investigated probiotics and prebiotics intervention in AD for preclinical and clinical trials, yet fewer studies have investigated the therapeutic effects of synbiotics. Synbiotics are a combination of probiotics and prebiotics. The metabolomic investigation revealed a notable influence of NMN synbiotics (β-nicotinamide mononucleotide (NMN), *L. plantarum*, and lactulose) on the gut metabolome, leading to a normalization of metabolic profiles in AD mouse models. The analysis additionally revealed that the differential metabolites exhibited enhanced functions within pathways associated with neurotransmitter synthesis and energy metabolism. This finding implies that NMN synbiotics may play a potential therapeutic role in modulating the GBA and synaptic function in AD [114]. The application of NMN synbiotics led to a notable decrease in the accumulation of Aβ in both the cerebral cortex and hippocampus, demonstrating reductions of 67% and 60%, respectively [66,67]. Additionally, NMN treatment improved histopathological conditions in the colon, characterized by a reduction in crypt depth and a restoration of goblet cell populations. The levels of tight junction proteins, specifically Claudin-1 and ZO-1, were markedly increased, which contributed to the improved integrity of the intestinal barrier [115].

NMN synbiotics resulted in a significant reorganization of the gut microbiota, characterized by a reduced ratio of *Firmicutes*/*Bacteroidetes* in the AD mouse model, which implies a possible improvement in gut dysbiosis. Analysis of alpha diversity metrics demonstrated a decline in microbial diversity subsequent to the administration of NMN synbiotics, whereas evaluations of beta diversity indicated a transition towards a more balanced structure within the microbial community [114]. Specifically, various intestinal bacteria exhibited significant enhancements, and modifications in gut microbiota may affect intestinal metabolism, consequently influencing various pathways following synbiotics treatment [116]. NMN synbiotics have been observed to reduce the expression of pro-inflammatory cytokines, including IL-1β, IL-6, and TNF-α, while also lowering levels of reactive oxygen species (ROS), indicating a decrease in oxidative stress [115].

Studies have optimized synbiotics through in vitro simulations of the human gastrointestinal system and machine learning algorithms to produce specific brain-bioavailable metabolites [62,63]. These metabolites, such as polyphenolic compounds, possess the ability to cross the blood–brain barrier, consequently inhibiting the aggregation of amyloid plaques and tau fibril formation [51]. Notably, synbiotics designed to produce these metabolites have demonstrated anti-inflammatory properties and may play a role in the deceleration of AD progression by addressing various neuropathological elements [117]. Indeed, synbiotic intake has been shown to reduce both local and systemic inflammation in mouse models of AD, indicating that dietary approaches targeting the gut–brain axis could represent an effective method for mitigating the advancement of Alzheimer’s disease [118]. Furthermore, research has identified specific synbiotic-derived metabolites, such as 3-hydroxybenzoic acid and 3-(3′-hydroxyphenyl) propionic acid, that can penetrate the blood–brain barrier and impede amyloid plaque and tau fibril aggregation [51]. Moreover, synbiotics have demonstrated the ability to influence gut microbiota and stimulate the PPARs signaling pathway, resulting in diminished neuroinflammation and improved cognitive function in mouse models of AD [116]. The collective findings highlight the promise of synbiotics as a versatile therapeutic strategy for addressing AD by targeting multiple neuropathological elements, such as inflammation, amyloid accumulation, and the aggregation of tau fibrils.

Interestingly, the administration of synbiotics has been shown to notably enhance learning and memory capabilities by reducing the deposition of Aβ proteins. Furthermore, synbiotics can activate the peroxisome proliferator-activated receptor (PPAR) signaling pathway, leading to a marked reduction in neuroinflammation within the brains of APP/PS1 mice [116]. For example, specific synbiotic formulation containing *Clostridium sporogenes* (1 × 10^10^ CFU per day) and xylan (1%, *w*/*w*) administered over a 30-day period resulted in substantial improvements in cognitive function and spatial memory in the 5×FAD transgenic model of AD. Overall, the supplementation with synbiotics has been found to significantly mitigate cognitive and intellectual impairments in 5×FAD mice, which may be partially linked to an increase in the production of indole-3-propionic acid (IPA) by the gut microbiota [119].

Overall, synbiotic formulations provide advanced bioavailability of microbially produced anti-inflammatory and antioxidant metabolites. This mechanism offers biotherapeutic potential that may enhance the benefit of microbiome modulation for the host [120]. Also, previous reports demonstrated the superior benefit of synbiotics compared to individual probiotics and prebiotics. Unfortunately, there is limited evidence to demonstrate the beneficial effects of synbiotics in slowing AD progression as inconsistent results in preclinical studies are observed. Some potential confounding factors that could affect gut microbiota composition include host diet, duration of intervention, and specification of microbial strains [9]. Hence, additional animal and human intervention trials need to be conducted to be focused on understanding the synergistic mechanism of action.

#### 5.2.4. Postbiotics

Functional research of postbiotics has emerged due to its potential to provide neuroprotective effects by mitigating inflammation, oxidative stress, and the aggregation of proteins linked to neurodegenerative processes. Furthermore, they may also promote beneficial neurological outcomes by increasing dopamine levels, protecting dopaminergic neurons, and enhancing BDNF secretion [121]. Importantly the gut microbiome plays a crucial role in various health outcomes, as its imbalances are linked to numerous diseases, including neurodegenerative conditions [10]. In this regard, strategies aimed at altering the microbiome through prebiotics, probiotics, and postbiotics have shown promise in alleviating symptoms associated with AD, and utilizing tyndallized *Bifidobacterium longum* and *Lactobacillus acidophilus* has demonstrated efficacy in disaggregating amyloid-β/Aβ-40 aggregates through the chelation of Zn^2+^ and Cu^2+^ ions. This intervention led to a reduction in the expression of endogenous human APPTG protein and a decrease in mouse APP gene expression in the hemibrains. While exercise training affects the NF-kB signaling pathway that modulates immune responses, the postbiotic treatment further reduces APP gene expression, disaggregates existing amyloid-β plaques, and activates mitochondrial protein quality control in brain regions beyond the hippocampus. Together, these treatments effectively slow the progression of AD [122]. In both ectothermic and endothermic animals, postbiotics from *Aspergillus oryzae* have been shown to enhance thermal tolerance and reduce inflammation, leading to improved survival and milk production. These compounds offer diverse benefits, including enhanced growth performance, improved immune function, and modulation of gut microbiota [123]. Their safety and stability have attracted considerable attention which responds to the need for more animal and human studies to fill in the gaps on AD’s mechanisms of action.

Potential mechanisms include immune system regulation and interference with pathogen attachment to host cells. Limited research indicates that postbiotics release key bacterial components, such as lipoteichoic acids, peptidoglycans, or exopolysaccharides, which have significant immunomodulatory effects against pathogens [124]. Furthermore, postbiotics are proposed to induce changes in gut microbiome, associated with increased levels of innate and acquired immunity biomarkers [125]. The therapeutic potential of postbiotics is still largely unexplored, as this is an emerging field of research.

**Table 2:** Probiotic, prebiotic, synbiotic, and postbiotic intervention for AD in animal studies.

Agents	Model Investigated	Treatments	Administration	Main Observations	Reference
Probiotics	Two-month-old C57BL/6J mice	*B. breve MCC1274* 1 × 10^9^ CFU/6.25 mg/200 μL saline/mouse/day	Oral gavage for four months	A significant decrease in soluble Aβ42 concentrations within the hippocampus. Activated the Akt/glycogen synthase kinase-3β (GSK-3β) signaling pathway. Decreased microglial activation and an elevation in synaptic protein levels in the hippocampus.	[98]
Probiotics	2–3 months male and female C57BL/6J and App^NL-G-F^ mice	VSL#3^®^ 4 × 10^9^ CFU/day/25 g mice (*L. plantarum*, *L. delbrueckii* subsp. *Bulgaricus*, *L. paracasei*, *L. acidophilus*, *B. breve*, *B. longum*, *B. infantis*, and *S. salivarius* subsp. *Thermophilus*)	Drinking water for 8 weeks.	Probiotic feeding reduced A plaque load and improved memory in App^NL-G-F^ female mice. Probiotics decreased microgliosis and TNF-α levels in App^NL-G-F^ female mice with no effects in male mice. Probiotic feeding stimulated innate immune response changes in App^NL-G-F^ female spleens.	[107]
Probiotics	6-month-old senescence-accelerated-mouse-prone 8 (SAMP8) and senescence-accelerated-mouse-resistant 1 (SAMR1)	Probiotic-2 (P2, a probiotic mixture of *Bifidobacterium lactis* and *Lactobacillus rhamnosus*) and probiotic-3 (P3, a probiotic mixture of *Bifidobacterium lactis*, *Lactobacillus acidophilus*, and *Lactobacillus rhamnosus*) 1 × 10^9^ CFU/mouse/day	Drinking water for 8 weeks	A significant improvement in the cognitive functions of SAMP8 mice. Marked differences in the relative abundance of ten bacterial taxa among the four experimental groups. Affect the levels of serum short-chain fatty acids (SCFAs), particularly valeric acid, isovaleric acid, and hexanoic acid. A substantial decrease in neural damage, along with reductions in Aβ and Tau pathologies and neuroinflammation, in the brains of SAMP8 mice.	[99]
Probiotics	Male 9-month-old SAMP8 and SAMR1 mice	ProBiotic-4, a probiotic preparation composed of *B. lactis* (50%), *L. casei* (25%), *B. bifidum* (12.5%), and *L. acidophilus* (12.5%)	Drinking water for 12 weeks	Improvement in memory capabilities. Decrease in neuronal and synaptic injury and glial activation. Changes in the composition of microbiota in both the feces and brains of aged SAMP8 mice. Alleviated age-associated deficits in the integrity of the intestinal barrier and the blood–brain barrier. Reduction in interleukin-6 and tumor necrosis factor-α at both mRNA and protein levels. Diminished the levels of plasma and cerebral lipopolysaccharide (LPS). Lowered the expression of Toll-like receptor 4 (TLR4). Nuclear translocation of nuclear factor-κB (NF-κB) within the brain.	[100]
Probiotics	3xTg-AD mice	Lab4P probiotic consortium, composed of *Lactobacillus acidophilus* CUL21 (NCIMB 30156), *Lactobacillus acidophilus* CUL60 (NCIMB 30157), *Lactobacillus plantarum* CUL66 (NCIMB 30280), *Bifidobacterium bifidum* CUL20 (NCIMB 30153) and *Bifidobacterium animalis* subsp. *lactis* CUL34 (NCIMB 30172) delivering a daily dose of ~5 × 10^8^ CFU/mouse/day (human equivalent dose of ~5 × 10^10^ CFU/day)	Lyophilized preparation (mixed with feed) for 12 weeks and 24 weeks	Alleviated the deterioration associated with disease in novel object recognition and in the density of hippocampal neuron spines. The probiotic has an anti-inflammatory impact under conditions of metabolic stress.	[126]
Probiotics	Male and female TH-CRE rats expressing pseudophosphorylated tau	ProBiotic-4, a probiotic preparation composed of *B. lactis* (50%), *L. casei* (25%), *B. bifidum* (12.5%), and *L. acidophilus* (12.5%) 3 × 10^9^ CFU/mouse/day	Drinking water for 3 months	Probiotic supplementation rescues the spatial learning deficiency. Probiotic supplementation increased gut microbiome diversity and improved bacterial composition. Probiotic supplementation reduces systemic and neuroinflammation in tau rats.	[127]
Prebiotic	6–8 months old 5×FAD male mice	*Pseudostellaria heterophylla* polysaccharide (PH-PS) 100 mg/kg/day	Oral gavage for 33 days	Enhancements in learning and spatial memory functions. A reduction in amyloid β accumulation. A suppression of reactive glial and astrocytic activation in 5×FAD mice. PH-PS modified the intestinal microbiota composition by increasing the abundance of beneficial probiotic species, such as *Lactobacillus*, *Muribaculum*, *Monoglobus*, and the *Eubacterium siraeum* group. PH-PS was effective in restoring the integrity of the intestinal barrier and alleviating peripheral inflammation. Promoted the transition of M1 microglia and A1 astrocytes to their protective M2 and A2 phenotypes.	[78]
Prebiotics	Male 5×FAD-transgenic mice	MOS (0.12% *w*/*v* in the drinking water, with a purity ~85%; Yuansen Biological Technology Ltd., Xi’an, China) or SCFAs mixture (acetate 67.5 mM, propionate 40 mM, butyrate 25 mM; Yuanye Biological Technology Ltd., Shanghai, China)	Drinking water added MOS or SCFAs for 8 weeks.	Improvements in cognitive abilities and spatial memory. A reduction in anxiety and obsessive compulsive behaviors in the 5×FAD transgenic model of Alzheimer’s disease. A significant reduction in Aβ deposits in the cortex, hippocampus, and amygdala. Reestablished the equilibrium of brain redox status and alleviated neuroinflammatory processes.	[110]
Prebiotic	4-month-old *APOE*4 mice	Prebiotic inulin diet contained 8% fiber from inulin.	Fed prebiotic inulin or control diet at four months for 16 weeks	A recovery of alpha diversity alongside a significant decrease in the levels of *Escherichia coli* and inflammatory pathway responses. The impact of diet and sex appeared to be less pronounced in *APOE3* mice.	[104]
Synbiotic	Six-month-old male APP^SWE^/PS1^ΔE9^ (APP/PS1) double-transgenic mice	Nicotinamide mononucleotide (NMN) synbiotics, a combination of NMN, *Lactiplantibacillus plantarum* CGMCC 1.16089, and lactulose (NMN: 300 mg/kg/day, tz-3647; *L. plantarum*: 10^8^ CFU/mL; lactulose: 200 mg/kg/day)	Daily gavage supplementation for three months	Decrease in Aβ accumulation within both the cerebral cortex and hippocampus. Positively influenced the histopathological state of the colon. A significant increase in the expression of tight junction proteins, particularly Claudin-1 and ZO-1. A decline in the levels of proinflammatory cytokines, including IL-1β, IL-6, and TNF-α. A reduction in reactive oxygen species (ROS).	[115]
Synbiotic	7-Month-old male 5 × FAD mice	*Clostridium sporogenes* ((ATCC15579, 10^10^ CFU/day via gavage)) and xylan (1% *w*/*w*)	Drinking water for 30 days	A significant reduction in amyloid-β (Aβ) deposits in both the cortical and hippocampal areas of the brain. Restored the synaptic ultrastructure in the affected mice and alleviated neuroinflammatory responses. An increase in the levels of the microbial metabolite indole-3-propionic acid (IPA). Improved the relative abundance of IPA-producing bacteria, such as *Lachnospira* and *Clostridium*, while reducing the prevalence of dominant bacteria linked to AD, including *Aquabacterium*, *Corynebacterium*, and *Romboutsia*.	[119]
Synbiotic	Male APP^swe^/PS1^ΔE9^ double transgenic mice, aged two months	NMN (300 mg/kg/day), *Lactobacillus plantarum* (10^8^ CFU/mL), and lactulose (200 mg/kg/day)	Gavage for three months	Restructuring of the gut microbiota, evidenced by a diminished *Firmicutes*/*Bacteroidetes* ratio in the Alzheimer’s disease (AD) mouse model. An enhancement of specific metabolite functions within pathways associated with neurotransmitter production and energy metabolism. A significant reduction in amyloid plaques due to Aβ accumulation in the brains of AD mice treated with NMN synbiotics.	[114]
Synbiotic	8-week old 3xTg-AD mice	Red lentils and coated with a probiotic carrier (dark chocolate) containing SLAB51 (*Streptococcus thermophilus* DSM 32245, *Bifidobacterium lactis* DSM 32246, *Bifidobacterium lactis* DSM 32247, *Lactobacillus acidophilus* DSM 32241, *Lactobacillus helveticus* DSM 32242, *Lactobacillus paracasei* DSM 32243, *Lactobacillus plantarum* DSM 32244, *Lactobacillus brevis* DSM 27961) 2 × 10^11^ bacteria/kg/day of SLAB51.	Drinking water for 4 months.	Chronic consumption of synbiotic preparation (functional cookie) preserved cognition, reduced amyloid load, improved glucose and lipid homeostasis, and diminished oxidation and inflammation-related damages. The synergistic effect was indicated by significantly higher glucose insulinotropic polypeptide concentrations in the functional cookie group compared to probiotic group. Aβ1-42 is reduced in both the hippocampus and the cortex of AD mice supplemented with synbiotic.	[128]
Postbiotic	Male APP/PS1 transgenic mice (B6C3-Tg (APPswe, PSEN1dE9)85Dbo/Mmjax; APP/PS1^TG^)	FRAMELIM^®^ contains tyndallized *Bifidobacterium longum* and *Lactobacillus acidophilus*lysates, in addition vitamins A, B1, B3, B6, B9, B12 and omega 3 fatty acids in cod liver oil.	FRAMELIM^®^ was administered five times weekly (120 mg/day) for 20 weeks with rodent chow (SDS/VRF1(P)).	A decrease in the expression of genes associated with AD, particularly NF-kB. Effectively disaggregate amyloid-β/Aβ-40 aggregates by chelating Zn^2+^ and Cu^2+^ ions. A reduction in endogenous human APPTG protein expression and mouse APP gene expression in the hemibrains.	[122]

### 5.3. Evidence from Human Studies

Recent AD microbiome therapy studies suggest that the supplementation of probiotics may have beneficial effects on cognitive function and neurodegeneration in humans (Table 3). A meta-analysis of clinical trials revealed a modest yet statistically significant enhancement in Mini-Mental State Examination scores with probiotic use, though results were heterogeneous. It appears that probiotics exert their effects through modulation of the GBA, reducing inflammation and oxidative stress [129]. Specifically, specific strains, like *C. butyricum* and *A. muciniphila*, have shown promise for neurodegenerative diseases [103]. Furthermore, a systematic review and meta-analysis of randomized controlled trials demonstrated improvements in cognitive function, inflammatory markers, and lipid profiles among patients with AD, mild cognitive impairment, and Parkinson’s disease following probiotic intervention [130]. For instance, the probiotic *B. longum BB68S* has been reported to enhance cognitive abilities in healthy older adults without cognitive deficits, while also positively influencing their gut microbiota. This evidence underscores the use of probiotics as a promising approach for fostering healthy aging and advancing research in cognitive aging [131]. In summary, probiotics have been shown to enhance cognitive performance in patients with AD or mild cognitive impairment, likely through the reduction in inflammatory and oxidative biomarkers [131].

On the other hand, prebiotic supplementation has demonstrated potential in attenuating age-related neurodegeneration in animal studies, with promising improvements in cognitive function observed [132]. Nonetheless, further clinical trials are essential to confirm its effectiveness in human populations. For instance, a small, open-label investigation involving patients with Parkinson’s disease demonstrated that prebiotic fiber intervention was well-tolerated and associated with favorable modifications in gut microbiota composition, SCFAs, inflammatory markers, and neurofilament light chain levels [133]. It is likely that the mechanisms by which prebiotics may produce these effects likely involve the promotion of beneficial metabolites, such as butyrate, alongside the regulation of gut immunity and maintenance of mucosal homeostasis, which subsequently influence brain function [109]. While some research has shown promising results in animal models, with metabolites derived from synbiotics demonstrating anti-inflammatory and neuroprotective effects [51], the results from human clinical trials have been inconsistent. A systematic review and meta-analysis of randomized controlled trials, for instance, revealed an insufficient evidence base to endorse the clinical use of probiotics in dementia patients, even though some metabolic markers showed improvement [134]. These findings underscore the necessity for additional research, particularly well-structured, large-scale human clinical trials, to elucidate the mechanisms and therapeutic efficacy of probiotics and synbiotics in the context of neurodegenerative diseases.

Another research has explored the potential of microbiome-based interventions, particularly postbiotics, in addressing neurodegenerative disorders like AD and Parkinson’s. Postbiotics have demonstrated efficacy in modulating gut microbiota, mitigating neuroinflammation, and targeting cellular mechanisms involved in ND pathogenesis [135]. Postbiotics are believed to offer neuroprotection by elevating dopamine levels, reducing α-synuclein and amyloid β peptide plaques, and ameliorating motor deficits [121]. Furthermore, they also exhibit anti-inflammatory and antioxidant properties, promoting BDNF secretion and inhibiting apoptosis [121]. While human studies are limited, existing studies indicate beneficial outcomes on disease-specific symptoms, general health, and metabolic indicators.

Despite its beneficial effects, clinical trials in biotherapeutics have several drawbacks. First, the sample size of some studies was not large enough to create high statistical power and effect size [136]. Second, some clinical trials’ design did not adhere to general principles of randomized, double-blind, or control [9,136]. Third, it would be more conclusive to include key clinical indicators of diagnostic and therapeutic implication, fulling the primary neuropathologic criteria for AD [136]. Finally, there is no consistency concerning the modified bacterial phyla in AD patients. It is essential to determine whether the alterations in the gut microbiome can directly impact disease progression of AD from clinical trials. Also, there is limited knowledge on specific bacteria taxa alterations observed in AD patients across studies. This might be due to inadequate sample sizes and various compounding factors affecting gut microbiome composition, including diet and environmental exposure [137]. Overall, probiotics, prebiotics, synbiotics, and postbiotics interventions have shown promising beneficial neuroprotective effects for AD in both pre-clinical and clinical trials, opening new avenues for potential therapy in AD. Yet, larger-scale intervention studies need to be performed following the trials’ guidelines for testing therapeutic effects in AD.

**Table 3:** Clinical trials on probiotics intervention in healthy and AD patients.

Subjects	Treatments	Doses	Study Design	N	Duration	Effects Compared to Placebo	Reference
Healthy elders ≥ 65 years	*Bifidobacterium bifidum* BGN4 and *Bifidobacterium longum* BORI	1 × 10^9^ CFU/day	Randomized, double-blind, placebo-controlled, multicenter clinical trial	63	12 weeks	A significant reduction in the relative abundance of gut bacteria linked to inflammation by week 12. Greater improvements in mental flexibility evaluations and stress scores when compared to the placebo group. A marked increase in serum concentrations of brain-derived neurotrophic factor (BDNF).	[138]
Patients with mild cognitive impairment (MCI) aged from 65 to 88 years old	*Bifidobacterium breve* MCC1274 (A1)	2 × 10^10^ CFU/day	Randomized, double-blind, placebo-controlled trial	130	24 weeks	A significant improvement in cognitive function. Prevented the progression of AD. The overall composition of the gut microbiota did not show substantial changes due to probiotic intervention.	[139]
AD patients aged between 50 and 90 years	*Bifidobacterium longum* subsp. *infantis* BLI-02, *B. breve* Bv-889, *B. animalis* subsp. *lactis* CP-9, *B. bifidum* VDD088, and *Lactobacillus plantarum* PL-02	5 × 10^7^ CFU/capsule	Randomized, double-blind active-controlled trial	40	12 weeks	A 36% increase in serum BDNF levels. A reduction in IL-1β. An improvement in the activity of the antioxidant enzyme superoxide dismutase (SOD).	[140]
Healthy older adults without cognitive impairment	*Bifidobacterium longum* BB68S (BB68S)	5 × 10^10^ CFU/sachet	Randomized, double-blind, placebo-controlled trial	60	8 weeks	Improved the cognitive functions of participants, especially in immediate memory, visuospatial and constructional skills, attention, and delayed memory. An increase in the relative abundance of beneficial bacterial populations, including *Lachnospira*, *Bifidobacterium*, *Dorea*, and *Cellulosilyticum*. Decreasing the levels of bacteria linked to cognitive decline, such as *Collinsella*, *Parabacteroides*, *Tyzzerella*, *Bilophila*, unclassified_c_*Negativicutes*, *Epulopiscium*, *Porphyromonas*, and *Granulicatella*.	[131]
AD patients aged 50–90 years	*Lacticaseibacillus rhamnosus* HA-114 *or Bifidobacterium longum* R0175	7.5 × 10^9^ twice daily	Randomized, double-blind, placebo-controlled trial	90	12 weeks	Significant improvements in serum markers associated with inflammation and oxidative stress.	[141]
Aged between 55 and 80 years	*Streptococcus thermophilus* GH, *Streptococcus salivarius* GH NEXARS, *Lactobacilus plantarum* GH, *and Pediococcus pentosaceus* GH	10^6^ CFU/day	Randomized, double-blind, placebo-controlled trial with a cross-over design	91	12 weeks	No variations in scores were observed across all cognitive assessments.	[142]
Older adults aged 50–79 years	*Bifidobacterium breve* A1	2 × 10^10^ CFU/day	Double-blind, randomized placebo-controlled trial	80	16 weeks	Safe and effective approach for improving memory functions in individuals who are suspected of experiencing mild cognitive impairment (MCI).	[143]

## 6. Safety and Efficacy

The effectiveness of probiotics, prebiotics, synbiotics, and postbiotics in diverse health conditions has become one of the major concerns in treatment development. In end-stage renal disease patients, prebiotics have proven most effective in lowering inflammatory markers and uremic toxins, while probiotics alleviated gastrointestinal symptoms [144]. For diseases like ulcerative colitis, probiotics containing *Bifidobacteria* showed promise in treating active disease while, in the case of non-alcoholic fatty liver disease, probiotics demonstrated the highest efficacy, followed by synbiotics and prebiotics [145]. In pre-diabetes management, probiotics showed potential in decreasing HbA1c and improving post-load glucose levels, while synbiotics have demonstrated better effectiveness compared to probiotics alone in terms of glycemic control [146]. Despite these advances, current evidence regarding substantial improvements in glucose metabolism, lipid profiles, and body composition in pre-diabetes remains insufficient. Overall, these findings imply that probiotics, prebiotics, and synbiotics may offer therapeutic advantages in various health conditions, but more high-quality research is needed.

The efficacy of prebiotics for AD is assessed based on the capacity to either improve or prevent cognitive decline. Although the administration of prebiotics has shown promise as AD therapy, the bioavailability presents barriers to their efficacy. Prebiotic bioavailability and bioactivity depends on gut microbiota diversity and its metabolite productions [147]. Furthermore, this intervention could also potentially address behavioral and emotional changes [137].

In terms of safety, probiotics, prebiotics, and synbiotics have been generally safe for use during pregnancy and lactation, with only minor adverse effects reported [148]. Additionally, these supplements are considered safe for immune-compromised adults, with adverse events occurring less frequently compared to control groups. In pediatric populations, no significant safety concerns have been noted for children aged 0-18 years across various health conditions [149]. However, both studies acknowledged inconsistent and imprecise reporting of adverse events. A prior meta-analysis investigating the impact of these supplements on adiponectin and leptin levels reported no significant hormonal changes following probiotic use, underscoring the need for standardized adverse event reporting. Limitation also persists due to variations in strains, dosages, and study populations [150].

Probiotics, such as *Bifidobacterium* and *Lactobacilli*, and prebiotics like FOS and inulin, may provide various health benefits, including immune system modulation and potential cancer prevention, particularly when utilized in combination as synbiotics [92]. Despite their favorable profile, some concerns persist regarding the reporting of adverse events in clinical trials, emphasizing the need for comprehensive safety evaluations. Probiotics and prebiotics have also demonstrated promise in treating various conditions, including gastrointestinal diseases and metabolic disorders. They may modulate host immunity, protect against infections, and aid in managing inflammatory bowel disease and cancer therapy [93]. However, additional research is essential to gain a complete understanding of their long-term safety and effectiveness across diverse populations and health conditions. Previous adverse effects of probiotics reported in clinical studies including systemic infection, GI side effects, gene transfer from probiotics to normal microbiota, harmful metabolic effects, and immune system stimulation. Future probiotic therapy for AD requires development to tackle colonization resistance [151].

Albeit extremely rare, probiotic therapy has safety concerns for carrying and spreading antibiotic resistance genes, presenting potential virulence factors, developing bacteremia or fungemia [136], and potentially inducing D-lactate metabolic acidosis, intestinal bacterial overgrowth, gas, bloating, and brain fogginess [137]. ISAPP raised a concern about the safety of probiotics, especially for vulnerable populations. These concerns include antibiotic resistance gene transfer via transformation, drug interaction, and potential impact of gut microbiome changes induced by probiotics. For such concerns, long-term studies focusing on probiotic safety in populations at risk need to be performed [137].

Postbiotics present no risk of infection for vulnerable or immunocompromised individuals, including infants and those with impaired immune systems. Furthermore, their use alongside antibiotics does not pose the threat of transferring resistance genes to other microorganisms. Due to the absence of living organisms, postbiotics are relatively more stable, demonstrating increased resistance to oxygen, temperature, and other environmental conditions, thereby preserving the integrity of their active components [125]. Despite its largely unexplored therapeutic potential, postbiotics and para probiotics are considered to have favorable safety profiles since they do not contain live organisms. For such reason, these biotics could avoid risks associated with probiotics [137].

## 7. Current Limitations and Future Directions

### 7.1. Current Limitations

Despite current data suggesting the significant biotherapeutic potential of probiotics, prebiotics, synbiotics, postbiotics, and paraprobiotics, many constraints need to be overcome before being consumed by the patients. It is reported that the United States Food and Drug Administration (FDA) has approved list of probiotics considered safe for commercial use, yet it is purposed for supplements. Until now, the FDA has not approved medical claims for biotherapeutics reducing the risk of diseases or as treatments [120].

In pre-diabetes treatment, these interventions showed partial benefits in modulating gut microbiota, but evidence for significant metabolic improvements remains insufficient [146]. In the field of sports health, while some positive health effects were reported for athletes and active individuals, substantial evidence for performance enhancement is lacking [152]. When it comes to oral health, these interventions present potential therapeutic and preventive strategies against candidiasis, with biogenics emerging as a promising concept [153]. Across these diverse areas, while early findings are encouraging, further investigations are essential to substantiate these interventions’ mechanisms and healthy impacts thoroughly. Despite these advances, achieving a definitive cure for AD disease remains elusive. Current treatments primarily aim to alleviate symptoms rather than offer a cure [154]. The intricate interaction among these biological factors suggests that AD progression is a highly dynamic process, involving a combination of protective mechanisms that, under certain conditions, may lead to adverse effects.

Overall, for biotherapeutic like probiotics, prebiotics, synbiotics, postbiotics, and para probiotics to be regulated as pharmaceutical or biological products, further research needs to be conducted. These interventions need to meet the regulator standards for the safety, efficacy, and purity of the compounds for medical applications. While comprehending the mechanism of action underlying biotherapeutic effects for AD, the opportunity is developed to create safe treatment to maximize their potential to combat AD.

### 7.2. Future Implication

The prospective implications of utilizing treatment modalities for AD that incorporate probiotics, prebiotics, synbiotics, postbiotics, and para probiotics through the GBA opens promising new directions for therapeutic development. These strategies target gut microbiota modulation, with goals to reduce neuroinflammation, oxidative stress, and the accumulation of amyloid-beta-three pivotal factors in AD pathology. Each approach offers unique benefits in restoring gut microbiome balance and supporting brain health. Probiotics and prebiotics are key players in this balance, encouraging the production of neuroprotective metabolites such as SCFAs, and enhancing neurotransmitter synthesis. Synbiotics strengthen these effects synergistically, amplifying the anti-inflammatory and neuroprotective impact on the central nervous system. Additionally, postbiotics and para probiotics represent an innovative approach, utilizing stable, bioactive compounds derived from beneficial bacteria. These compounds are particularly valuable for their regulated anti-inflammatory and antioxidant properties, offering a precise and controlled treatment option. Together, these interventions may not only help improve cognitive function but also modify the disease’s progression, positioning them as powerful candidates in AD therapy. Furthermore, these strategies hold potential for advancing personalized medicine in AD. Tailoring these therapies based on an individual’s unique microbiome profile could lead to more effective and individualized treatment outcomes, marking a significant step forward in managing AD.

## 8. Conclusions

In summary, the GBA influences gut microbiota to mitigate the accumulation of neuroinflammation, oxidative stress, and amyloid beta in AD. Numbers of recent observational studies consistently indicate that the gut microbiome has a synergistic impact to decrease disease progression, opening a new paradigm for therapeutic intervention. The use of probiotics and prebiotics enhances the production of neuroprotective metabolites such as SCFAs and promotes the synthesis of neurotransmitters. The application of synbiotics offers a synergistic strategy to amplify these beneficial effects, while postbiotics and para probiotics provide greater control over therapeutic interventions with minimum risk. Meanwhile, postbiotics and para probiotics offer a novel level of therapeutic control, representing a new frontier in microbiome-based AD therapy by delivering specific, active metabolites without the variability seen in live bacterial therapies. Together, these microbiome-modulating therapies represent a comprehensive approach to AD treatment, with the potential to not only manage symptoms but also modify the disease course itself. These biotherapeutic treatments hold great promise in the upcoming treatment and prophylaxis of AD. Their impact has been demonstrated across preclinical studies and growing number of human clinical trials, underscoring their potential to influence cognitive function positively. As research advances, these interventions may lead to more personalized, targeted therapies, leveraging the GBA for enhanced AD management and potentially paving the way for broader applications in neurodegenerative treatment. However, significant efforts are still required to address fundamental unanswered questions concerning the role and mechanisms of the gut microbiota in AD, develop standardized protocols for gut microbiome research, and produce high-quality data from both preclinical and clinical studies, ultimately facilitating the translation of gut microbiome research into clinical practice.

## Figures and Tables

**Table 1:** Pharmacological treatment of AD.

No	Pharmacological Therapy	Dosage	Mechanism(s) of Action	Side Effect/Limitation	References
1.	Monoclonal antibody - Donanemab - Aducanumab - Lecanemab	The initial three doses should be administered at a dosage of 700 mg, followed by a subsequent dosage of 1400 mg, delivered intravenously every four weeks.	A humanized monoclonal antibody of the immunoglobulin gamma 1 (IgG1) class that targets insoluble N-truncated pyroglutamate amyloid beta.	Amyloid-related imaging abnormalities or ARIA, headache, reactions associated with infusions.	[37]
2.	Anticholinesterase (AChE) inhibitors - Donepezil - Galantamine - Rivastigmine	The starting dosage is 5 mg administered once daily, which may be increased to 10 mg once daily, the highest recommended dosage, after a period of 4 to 6 weeks.	Elevating acetylcholine concentrations in the brain is crucial, as this molecule facilitates the communication of information among specific neurons and is integral to memory processes. Furthermore, acetylcholinesterase (AChE) inhibitors promote cholinergic neurotransmission by obstructing the hydrolysis of acetylcholine, thereby leading to an increase in its synaptic availability.	The likelihood of experiencing secondary adverse effects is heightened, while the effectiveness of the medication is diminished.	[38]
3.	Anti-glutaminergics - Memantine	The starting dosage is 5 mg administered once daily, with a maximum recommended dosage of 20 mg to be considered after a duration of 4 weeks.	Modulate glutamate concentrations by employing a noncompetitive antagonist action on NMDA receptors. The NMDA receptor blockade inhibits the entry of intracellular calcium (Ca^2+^), thereby mitigating excitotoxicity that leads to neuronal degeneration in AD.	The effectiveness of interventions is, at best, limited and transient, addressing only the outcomes of Alzheimer’s disease rather than its underlying causes.	[39]
